# Wilhelm Hasselbach: a personal tribute

**DOI:** 10.1007/s10974-016-9449-1

**Published:** 2016-07-18

**Authors:** Johann Caspar Rüegg

**Affiliations:** University of Heidelberg, Heidelberg, Germany

**Keywords:** Sarcoplasmic reticulum, SERCA, Calcium pump

## Abstract

Wilhelm Hasselbach (1921–2015) is best known for his major contribution to the discovery and molecular elucidation of an ATP-driven active ion transport across a biological membrane. He had discovered SERCA, the calcium pump of the sarcoplasmic reticulum (Hasselbach and Makinose in Biochem Z 333:518–528, 1961; Biochem Z 339:94–111, 1963).

**W**ilhelm Hasselbach (1921–2015) was born in Falkenstein, a small village north of Frankfurt/Main, Germany. For most of his life he worked and lived in Heidelberg, where he was a director at the Max Planck Institute for Medical Research. He is probably best known for the discovery of the *calcium pump* of the sarcoplasmic reticulum (Hasselbach and Makinose 1961, 1963), which is known to be responsible for muscle relaxation. To be sure it was agreed then that the particulate (vesicular) fraction of the fragmented sarcoplasmatic reticulum was a “*relaxing factor*” (c.f. also Ebashi and Lipmann 1962). But before 1961, it was not at all clear whether the vesicular particles (Hasselbach’s “relaxing granules”, c.f. Nagai et al. 1960) produced a soluble relaxing factor inhibiting the interaction of actin and myosin or whether they “acted by binding calcium”, as already suggested by Annemarie Weber (quoted by Dorothy Needham in her seminal book “Machina Carnis”). And indeed, “free” calcium in the medium was found to be *actively* removed by the particulate “factor”—in fact by transporting calcium ions across an osmotic gradient into the vesicles of the particulate fraction, as beautifully demonstrated by Hasselbach and Makinose (1960, 1961). About the same time, Setsuro Ebashi (1961) also found that the “relaxing material” of muscle removed calcium from the medium in vitro. His independent finding was, however, not fully published until the following year (Ebashi and Lipmann 1962).

Clearly Hasselbach’s discovery paved the way for the later detailed elucidation of active calcium transport mechanisms and their role in the relaxation of skeletal, smooth and cardiac muscle (c.f. Hasselbach 1964; Hasselbach and Oetliker 1983). For instance, Hasselbach and Makinose (1962) were able to show that calcium transport and (Ca^2+^-activated) ATP splitting, as well as the so called ATP–ADP exchange reaction, were tightly coupled, suggesting the transient formation of an intermediate phosphorylation of a putable calcium transporter protein. Also, this process was found to be higly efficient: for every molecule of ATP split two ions of calcium were actively transported across the membrane surrounding the vesicles of the reticulum. Furthermore, by manipulating the experimental conditions, the calcium pump could be reversed, resulting in ATP synthesis rather than in breakdown, as well as in passive calcium efflux from the calcium storing vesicles. Thus Makinose and Hasselbach (1971) arrived at the conclusion “that the calcium translocation across the sarcoplasmic membranes is reversibly connected with the phosphoryl transfer reaction giving rise to a splitting of ATP when calcium moves inward and to an ATP synthesis when calcium moves outward”.

As later noted by Hasselbach and others, several drugs, including caffeine, also cause calcium efflux, but this was not found to be related to the reversal of the pump but rather to the passive release of stored calcium through calcium ion permeable channels, which could be blocked by ryanodine. Later experimental studies by Hasselbach and his long-term collaborator Andrea (“Andy”) Migala (1992) confirmed “that the caffeine sensitive calcium channel functions as an assembly of at least four ryanodine binding sites whereby the occupation of one site suffices to abolish [caffeine induced] calcium release”. This, of course, fitted well into current schemes of the role and mechanism of ryanodine receptors in excitation contraction coupling.

While these findings were interesting, Hasselbach’s most outstanding achievement was certainly his major contribution to the discovery and molecular elucidation of an ATP-driven active ion transport across a biological membrane. *He had discovered the Ca* ^*2*+^-*ATPase (SERCA) of the sarcoplasmatic reticulum*! Surely this success ought to be seen in the context of Jens Christian Skou’s discovery (Nobel Prize in 1997) of the Na/K-ATPase in 1957, which was then proposed to be *possibly* related to the active ion transport across the nerve membrane (Skou 1960). And most importantly, Peter Caldwell and colleagues (Caldwell et al. 1960) already suggested that “the *presence* of ATP may be required for the normal operation of (an) active transport mechanism”! Because of his numerous achievements, Wilhelm Hasselbach was awarded the prestigious Feldberg Prize in 1963, and the Paul Morawitz Prize of the German Society of Cardiology in 1986. He was elected a member of the German Academy of Science Leopoldina (Deutsche Akademie der Naturforscher Leopoldina) in 1969, a member of the Brasilian Academy of Science in 1975, and an honorary member of the German Physiological Society in 1991. In 1961, Hasselbach became a scientific member of the Max Planck Society, and as from 1966, he was a director at the Heidelberg Max Planck Institute for Medical Research until his retirement in 1989. Besides, he was an honorary professor at Heidelberg University whose lectures on applied physiology were highly appreciated by medical students.

**B**y all standards, Professor Hasselbach was a kind, helpful and modest man. Moreover he was always grateful for the help he received from others, and in particular in those difficult times after World War II when he was wounded. In 1998, looking back to his life-long experimental work, he stated: “The discovery of the ATP-driven calcium pump in the sarcoplasmic reticulum membranes reaches back to the postwar [World War II] years and would not be possible without the generous support by the American scientific community…; these pre- and postwar relations helped to establish the calcium pump as a physiologically relevant mechanism in all kinds of cells” (Hasselbach 1998).

Ironically, Hasselbach had his first opportunity to visit the United States rather late. John Gergely had invited him, H. H. Weber and me to a meeting taking place in May 1962 on the occasion of the opening of the Retina Foundation Institute in Boston. We all met at Dedham, Massachusetts, in a luxurious New England mansion, together with other visitors from Japan, England, Canada, Italy, Belgium, Sweden and Poland, where we were kindly received by young and old members of the American muscle community. We had long and unrestricted discussions, and those on the nature of the “relaxing factor” were somewhat heated. It was even proposed that the calcium pump could not cause relaxation of contractile systems per se. Rather it was suggested that the “true” relaxing factor might be a soluble inhibitor released from the sarcoplasmic reticulum but inactive unless myoplasmic free calcium was removed by the pump. In general, Hasselbach was a remarkable discussant. As Avril Somlyo once recalled, “he had an intensity and enthusiasm that made him special to interact with… always good discussions”!

After the Dedham meeting, Hasselbach and I visited Annemarie Weber’s Lab in New York. He then visited Fritz Lipmann, and I went on to work for a couple of weeks with David Bohr in Ann Arbor, Michigan. Soon the concept of the “soluble relaxing factor” was abandoned when Annemarie Weber found that many phenomena concerning the inhibition of actomyosin ATPase and of contraction by the tentative factor could be explained simply by the removal of calcium ions contaminating crude preparations of actomyosin and myofibrils (Weber et al. 1963), which on the other hand could be activated by traces of free calcium binding cooperatively to the myofibrillar proteins (Weber and Herz 1961). As later pointed out by Hasselbach (1989), “the ‘soluble relaxing factor’ finally became obsolete, when Ebashi (1963), Ebashi et al. (1967) showed that the ‘contractile proteins’ themselves contain a complex proteinsystem (originally called “native tropomyosin” or simply “Ebashi factor”) which in the absence of calcium prevents the interactions between ATP, myosin and actin”.

In retrospect, the meetings in Boston and Dedham were most memorable events for both of us. In fact this was our first journey to the United States—both of us travelling together on the old ocean liner “Hanseatic”. Actually I knew Wilhelm (“Willi”) Hasselbach long before this, in fact already since the summer of 1957, when he visited Dorothy Needham, Samuel Victor Perry and Kenneth Bailey, my research supervisor in Cambridge. I was then working on catch muscles of clams, and Hasselbach kindly showed me how to prepare skinned (i.e. glycerinated) smooth muscle fibre bundles and possibly use them to study contraction. And then he added, “perhaps later in Heidelberg…”, and indeed I became his colleague later on. Naturally, Hasselbach was *the* expert as he had just shown that, unlike skeletal muscle, skinned smooth muscle preparations had a rather high magnesium requirement for contraction (Hasselbach and Ledermair 1958). Hasselbach was then already well known to the muscle community, as he had devised a method (the famous “Hasselbach-Schneider solution”) by which myosin could be selectively extracted from skeletal muscle, thus leaving actin behind. Remember, his method had allowed Jean Hanson and Hugh Huxley to show that the myosin component of muscle resided exclusively in the A band, the length of which stayed constant in muscle contraction and relaxation as proposed by the sliding filament hypothesis. This in fact is a telling story that I knew first “from hearsay”, and much later also from Hasselbach’s personal account in a book chapter (Hasselbach 1989).

In 1949, and after completing his doctoral thesis in medicine, Hasselbach joined the Team of H. H. Weber in Tübingen, where he met Hildegard Portzehl, Weber’s daughter Annemarie, and Gerhard Schneider. Together with Schneider, he was meant to extract globulin X, which Weber had described in the thirties as a major protein constituent of muscle. Much to the dismay of “boss” Weber, they did not succeed. Instead they observed that myosin was selectively extracted from striated muscle such that its band structure disappeared. Importantly, they used salt solutions containing pyrophosphate substituting for the then barely available ATP (Hasselbach and Schneider 1951). These solutions were later called Hasselbach-Schneider solution by Jean Hanson and Hugh Huxley, when they used it to selectively remove myosin from the A band of myofibrils. With this work and the subsequent electron microscope studies, Hasselbach earned international recognition (Hasselbach 1953). It was Jean Hanson who took the initiative to promote further collaboration and visited him in early 1954, shortly after the Weber group had moved to the Max Planck Institute in Heidelberg.

Wilhelm (Willi) Hasselbach was a devoted scientist but also a kind-hearted family man. Friends of the family wrote, “he seemed like a great person and father”. Wilhelm Hasselbach died in Heidelberg at age 94. As he had wished, he was buried in Falkenstein, the village where he was born.
